# Dynamic Alterations of Oral Microbiota Related to Halitosis in Preschool Children

**DOI:** 10.3389/fcimb.2021.599467

**Published:** 2021-02-26

**Authors:** Yu Zhang, Ce Zhu, Guizhi Cao, Jingyu Zhan, Xiping Feng, Xi Chen

**Affiliations:** ^1^Department of Preventive Dentistry, Shanghai Ninth People's Hospital, College of Stomatology, Shanghai Jiao Tong University School of Medicine, Shanghai, China; ^2^National Clinical Research Center for Oral Diseases, Shanghai, China; ^3^Shanghai Key Laboratory of Stomatology and Shanghai Research Institute of Stomatology, Shanghai, China

**Keywords:** halitosis, oral microbiome shift, preschool children, longitudinal study, 16S rRNA gene, halitosis-onset prediction model

## Abstract

**Objective:**

This longitudinal study was aimed to evaluate the dynamic shift in oral microbiota during the process of halitosis progression among preschool children.

**Methods:**

The oral examinations, questionnaires and tongue coating specimens were collected at the baseline and 12-month follow-up. All children were oral healthy at the enrollment. At the 12-month follow-up, children who developed halitosis were included to the halitosis group (n = 10). While children who matched the age, gender, kindergarten and without halitosis were included to the control group (n = 10). 16S rRNA gene sequencing was used to reveal the shift of the tongue coating microbiome in these children during the 12- month period with the Human Oral Microbiome Database.

**Results:**

A remarkable shift in relative abundance of specific bacteria was observed prior to halitosis development. The principal coordinates and alpha diversity analyses revealed different shifting patterns of halitosis and the healthy participants’ microbiome structures and bacterial diversity over the 12-month follow-up. Both groups showed variable microbiota community structures before the onset of halitosis. Halitosis-enriched species *Prevotella melaninogenica*, *Actinomyces* sp.*_HMT_180* and *Saccharibacteria TM7_G-1_bacterium_HMT_352* were finally selected as biomarkers in the halitosis-onset prediction model after screening, with a prediction accuracy of 91.7%.

**Conclusions:**

The microbiome composition and relative abundance of the tongue coatings in the halitosis and control groups remarkably differed, even prior to the onset of the clinical manifestations of halitosis. The halitosis prediction model constructed on the basis of tongue coating microbiome biomarkers indicated the microbial shifts before the halitosis onset. Therefore, this can be considered for the timely detection and intervention of halitosis in children.

## Introduction

Halitosis is characterized by an offensive and unpleasant odor originating from the oral or nasal cavity, and pharynx (Cortelli et al., 2008). Several studies have demonstrated the high prevalence of halitosis, which is a public health concern that affects quality of life, social embarrassment and psychological restrictions (Aimetti et al., 2015; Chen et al., 2016). Children are in a critical period of personality development and building of social relationships. Restrictions in social activities caused by halitosis may influence a child’s psychological development.

Approximately 90% of all types of halitosis originates from the oral cavity, (intra-oral halitosis) (Cortelli et al., 2008). The most common intra-oral causes of halitosis include poor oral hygiene, bacterial coating of the tongue, stomatitis, xerostomia, or chronic bacterial infections such as caries, gingivitis, and periodontitis (Bornstein et al., 2009). In addition, age and dental caries may also contribute to halitosis in children (Nalcaci and Sonmez, 2008). Intra-oral halitosis is directly correlated to the formation of volatile sulfur compounds (VSCs), which include hydrogen sulfide (H_2_S), methyl mercaptan and dimethyl sulfide. Gram-positive and Gram-negative bacteria in the oral cavity can biodegrade sulfur-containing amino acids to produce VSCs and unpleasant odors (Kostelc et al., 1981; Persson et al., 1990; Goldberg et al., 1994; Loesche and Kazor, 2002; Sterer et al., 2009).

Several studies have analyzed the oral microbiota of halitosis and healthy adults, and reported remarkable variations in terms of microbiome composition and richness (Yang et al., 2013; Ren et al., 2016a; Seerangaiyan et al., 2017). Furthermore, longitudinal cohort studies have determined the process of halitosis development in terms of microbiome changes (Teng et al., 2015; Xu H et al., 2018; Dashper et al., 2019). Present literature reporting on various aspects of halitosis in preschool children remains scarce. Hence, understanding the development and behavior of the oral microbiome over time among healthy and halitosis preschool children may assist in the identification of children who are prone to develop halitosis. Furthermore, the early detection of halitosis development may help clinicians effectively intervene at the critical time, and prevent the development and further progression of halitosis.

The present longitudinal study investigated the dynamic changes in the tongue coating microbiome of preschool children at two time-points by utilizing the Illumina Miseq Sequencing of the 16S rRNA gene. The aim of the present study was to assess the dynamic shift in microbial composition of halitosis-associated bacterial species during the process of disease progression in preschool children. In addition, the possibility of using tongue coating microbial profiles for the early-stage diagnosis of halitosis was investigated, which may limit the extent of halitosis and further improve overall quality of life.

## Materials and Methods

### Subjects and Study Design

The present study recruited junior class children (age: three years old; *n* = 260) from three kindergartens (two from the suburbs and one from the central region) in Shanghai, China. Prior to enrollment and routine oral examination, the parents or guardians of these recruited children provided a signed informed consent. The research protocol was approved by the Ethics Committee at the Ninth People’s Hospital, School of Medicine, Shanghai Jiao Tong University, Shanghai, China (Ref#: 2015135). All children underwent comprehensive clinical oral examinations, and the participant’s guardian was instructed to complete the questionnaire survey at the time of enrollment (zero month), and at the 12-month follow-up.

The participants shared similar living conditions (all-day care kindergarten) throughout the study period, and had the same oral health habits and nutrition during the daytime, while staying in the kindergartens. During the 12-month follow-up, specially trained dentists periodically visited the kindergartens, and performed clinical examinations, halitosis assessments and oral sample collection.

All clinical assessments were carried out in the morning. In order to diagnose the halitosis, the organoleptic assessment (OS) criteria was applied, as previously described (Rosenberg et al., 1991). A well-trained and experienced dentist (Y.Z.) evaluated the OS scores at the baseline and follow-up visits, who was trained by the smell identification tests (Sensonics Inc., Haddon Heights, NJ, USA) to distinguish odors. The examiner was evaluated for detecting the olfactory sensitivity using a series of gradient concentrations of isovaleric acid, skatole, putrescine, and dimethyl disulfide. Children who had an OS score of 2 or more were classified to have halitosis. A trained and licensed dentist assessed the status of participants’ oral health including the dental caries, periodontium, plaque accumulation, and tongue coating. In addition, the plaque index (PLI), decayed, missing, and filled tooth (dmft) and decayed, missing, and filled surface (dmfs) indices were also calculated. Evaluation of the tongue coating was estimated based on its area and thickness as described previously (Seerangaiyan et al., 2018). The corresponding examination was performed by the same dentist at the baseline and follow-up visits. To assess intra-examiner reliability, 10% of subjects were chosen randomly and reexamined. The Cohen’s kappa (κ) values for all clinical measurements (0.83–0.98) demonstrated a good reliability.

At the baseline, the following exclusion criteria was applied to the participants: (1) refusal to participate or sign the informed consent; (2) OS >0; (3) dmfs >0; (4) mean PLI >1; (5) presence of mucosal or any systemic disease; (6) history of using antibiotics within the last three months. At the follow-up, the following inclusion criteria were applied: (1) dmfs=0; (2) mean PLI ≤ 1; (3) no mucosal disease and systemic disorders; (4) no history of using antibiotics during the last 3 months.

**Figure 1:**
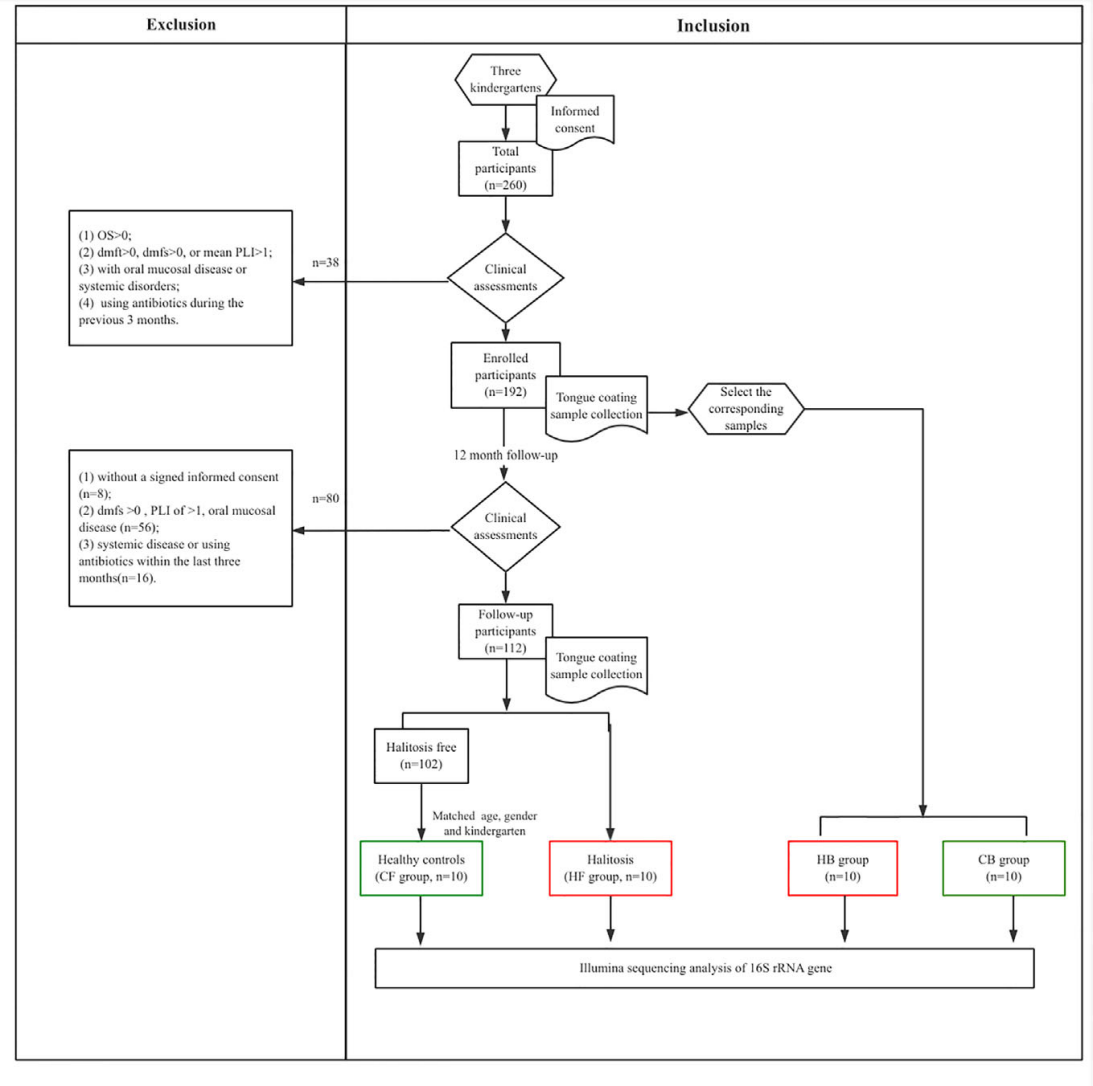
The flow chart representing the study selection criteria and recruitment of participants for the subsequent analysis.

After the clinical examination of 260 children, 192 children who fulfilled the selection criteria were recruited at baseline. At the 12-month follow-up, 56 children were excluded for having oral diseases, while 16 children were excluded for reporting a history of systemic disease or antibiotics usage within the last three months. Another eight children refused to participate due to personal reasons. Among the remaining children (*n* = 112), 10 children reported newly developed halitosis, and were included to the halitosis group. Furthermore, another 10 healthy children, with matched the age, gender and kindergarten, but without halitosis, were recruited to the control group (**Figure 1**).

### Sample Collection, DNA Extraction, and Sequencing of Tongue Coating

All participants were instructed to refrain from consuming any drink or food at least 2 h before the tongue coating specimen sample collection. The participant’s tongue was retracted up from the floor of the mouth, and the tongue coating was gently scraped from the dorsal to ventral areas using a sterile tongue cleaning device. All specimens were taken in the morning (9–11 a.m.). All collected specimens were stored in a container with dry ice, and transferred to a freezer (-80°C) within 4 h of sample collection. A soil DNA Kit (EZNA^®^; Omega Bio-tek, Norcross, GA, USA) was used to extract the microbial DNA of the tongue scrapped coating. The purity and final concentration of the DNA was assessed using a UV-vis spectrophotometer (NanoDrop2000; Thermo Scientific, Wilmington, MA, USA). The qualitative analysis of the DNA was performed by agarose gel (1%) electrophoresis. The amplification of the bacterial 16S-rRNA gene (V3-V4 hypervariable segments) was performed using the thermocycler PCR system (GeneAmp 9700, ABI, USA) and the associated primers (338F: 5’-ACTCCTACGGGAGGCAGCAG-3’; 806R: 5’-GGACTACHVGGGTWTCTAAT-3’). The whole PCR was completed, as previously described (Huws et al., 2007). Briefly, the specimens were denatured (3 min; 95°C, 27×30 second cycles at 95°C), annealed (30 s, 55°C) and elongated (45 s, 72°C), and subject to a final extension (10 min, 72°C). Aliquots of 20 μl (containing 4 μl of 5×FastPfu Buffer, 2 μl of 2.5 mM dNTPs, 0.8 μl of each primer [5 μM], 0.4 μl of Fast Pfu polymerase, and 10 ng of the DNA template) were used to run the experiments in triplicate. Furthermore, agarose gels (2%) and the AxyPrep DNA Gel Extraction Kit (Axygen Biosciences, Union City, CA, USA) were used to extract the PCR products that were further purified by QuantiFluor™-ST (Promega, Fitchburg, WI, USA). After the purification, the amplicons were pooled in the equimolar paired-end sequence (2 × 300) using the Illumina MiSeq platform (Illumina, San Diego, CA, USA), according to the guidelines specified by Majorbio Bio-Pharm Technology Co. Ltd. (Shanghai, China). The raw sequencing data of the present study is accessible from the NCBI Sequence Read Archive (Accession# PRJNA632355).

### Bioinformatics and Statistical Analyses

According to the RDP Classifier algorithm (http://rdp.cme.msu.edu/), each 16S-rRNA gene sequence was classified and analyzed against the Human Oral Microbiome Database (HOMD) database (v15.2). The representative sequences were classified according to various phylum and species levels. The sequencing data were interpreted by the Trimmomatic and FLASH software (Caporaso et al., 2010; Magoc and Salzberg, 2011), while the UPARSE v7.1 software (http://drive5.com/uparse/) (Schloss et al., 2009) was used to cluster the operational taxonomic units (OTUs) (similarity cutoff rate: 97%). The UCHIME (version 4.1) was used to identify and extract the chimeric sequences.

The bacterial richness diversity of the tongue coating microbiota was assessed using the alpha (α) index. Principal coordinate analysis (PCoA) was carried out using QIIME, based on the normalized weighted Unifrac distance matrices (Caporaso et al., 2010). The present study performed the analysis of similarities (ANOSIM) to evaluate the tongue microbiota composition of the participants. Hierarchical clustering was conducted using the unweighted pair-group method with arithmetic means (UPGMA) (Ramette, 2007). The relative abundance (mean differences) in species was evaluated by linear discriminant analysis (LDA) effect size (LEfSe) (Segata et al., 2011), and a fixed alpha value (0.05) was used for the Kruskal–Wallis test. A threshold of 2.0 was used to analyze the logarithmic LDA scores. The functional profiling of microbial communities was predicted using the phylogenetic investigation of communities by reconstruction of unobserved states (PICRUSt) (Langille et al., 2013), followed by collapsing the predicted functional composition profiles into level 3 of the Kyoto Encyclopedia of Genes and Genomes (KEGG) database pathways (Tang et al., 2018).

The data was analyzed by SPSS (v20, IBM, NY, USA) using a significance level of 5%. Cohen’s κ test was used to measure the intra-examiner reliability of the examiners. Descriptive statistics were applied to analyze the sociodemographic and clinical characteristics of the participants. Student’s t-test was applied to analyze the differences, in terms of continuous variables, comparing the alpha diversity, and predicting the metabolic pathways of the halitosis and control groups. Furthermore, Chi-square test was applied to compare both groups based on lifestyle and clinical factors. The halitosis-onset prediction model was constructed using logistic regression.

## Results

### Demographic Characteristics and Questionnaire Data

The final analysis and 16S rRNA gene sequencing of the tongue coating samples was carried out for 20 subjects, which included the halitosis and control groups (*n* = 10 for each group). All samples were compared in the four subgroups: halitosis group at baseline (HB, 10 subjects), halitosis group follow-up (HF, 10 subjects), control group at baseline (CB, 10 subjects) and control group at follow-up (CF, 10 subjects). The participants in the halitosis and control groups exhibited no significant differences in terms of gender (*P* = 1.000), age (*P* = 0.545), and main demographic and socioeconomic characteristics such as body mass indices (*P* > 0.05, for all). The participant of both groups showed no significant difference in their PLI and tongue coating (*P* > 0.05). Furthermore, there was no significant difference in both groups, in terms of oral hygiene habits (tooth brushing frequency, parental assistance for brushing teeth, and frequency of oral examination), and dietary habits (such as meat consumption, and frequency of consuming fruits or sweets) (**Appendix Table S1**, *P* > 0.05 for all).

### Sequencing Data

The present study investigated 40 samples that generated a total of 1,810,179 high-quality reads, with an average of approximately 45,254 sequences per sample. The average length was 433. The sequence OTU clustering and notation (at a 3% divergence level) identified a total of 11 phyla, 26 classes, 48 orders, 83 families, 152 genera, 314 species, and 657 OTUs. The rarefaction indicated the near-complete sampling of the tongue coating community (**Appendix Figure 1A**). The number of shared and unique OTUs for the four subgroups are presented in **Appendix Figure 2B**.

### The Longitudinal View of Tongue Coating Microbiota Dynamics

**Appendix Figure 2** presents the distribution of bacterial relative abundances from the phylum to species level. There were 64 families, 114 genera, 242 species, and 446 OTUs at baseline, and these increased to 79 families, 140 genera, 293 species, and 609 OTUs upon follow-up.

**Figure 2:**
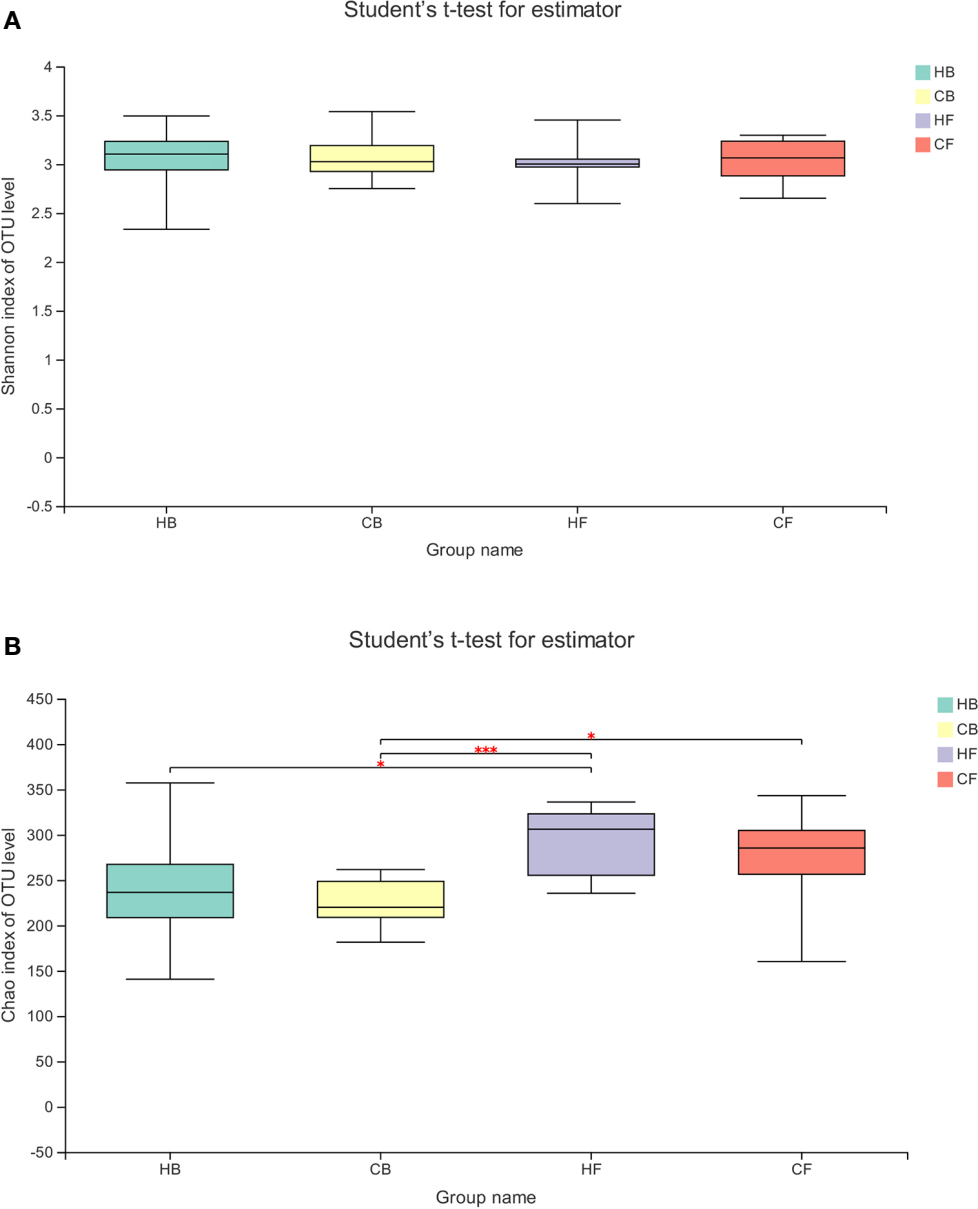
The temporal dynamics of microbiota alpha diversity. **(A)** The Shannon index; **(B)** the Chao index; ^*^P < 0.05, ^***^P < 0.001.

The alpha diversity of the tongue coating bacterial microbiome was evaluated using the richness index (Chao) and diversity index (Shannon). The Shannon index had no significant differences between groups and within groups (**Figure 2A**, HB *vs.* HF *P* = 0.691, CB *vs.* CF *P* = 0.645, HB *vs.* CB *P* = 0.937, HF *vs.* CF *P* = 0.898, respectively). The comparative evaluation of the Chao index indicated that the follow-up samples in both the halitosis and control groups have a significantly higher microbial diversity index, when compared to the baseline samples (**Figure 2B**, HB *vs.* HF *P* = 0.046, CB *vs.* CF *P* = 0.024, respectively). However, the Shannon index exhibited a decrease tendency in the halitosis group, although the difference was not significant.

**Figure 3:**
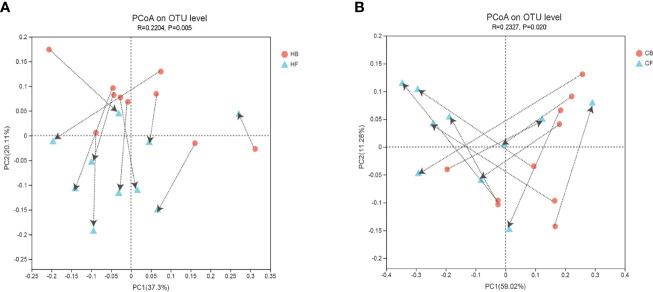
Intra-individual shifts of the microbiome community structure using the principle coordinates analysis (PCoA), based on the weighted normalized UniFrac distance, in comparing the shifts in community structure, from baseline to the 12-month follow-up. **(A)** The halitosis group; **(B)** The control group.

The PCoA based on the weighted normalized UniFrac distance validated the intra-individual variations in the structure of tongue coating microbiota in each sample during halitosis development and normal growth (**Figure 3**). The results indicated that the microbiome in these two groups demonstrated evident shifting patterns over a period of 12-months. The majority of the samples in the HB group shifted in the same direction, and the PC values in nine of 10 samples decreased during the follow-up (**Figure 3A**). Compared to the halitosis groups, the healthy participants exhibited irregular variations in microbiome structures while growing up (**Figure 3B**).

### Early Changes in the Tongue Coating Microbiome and Halitosis

**Figure 4:**
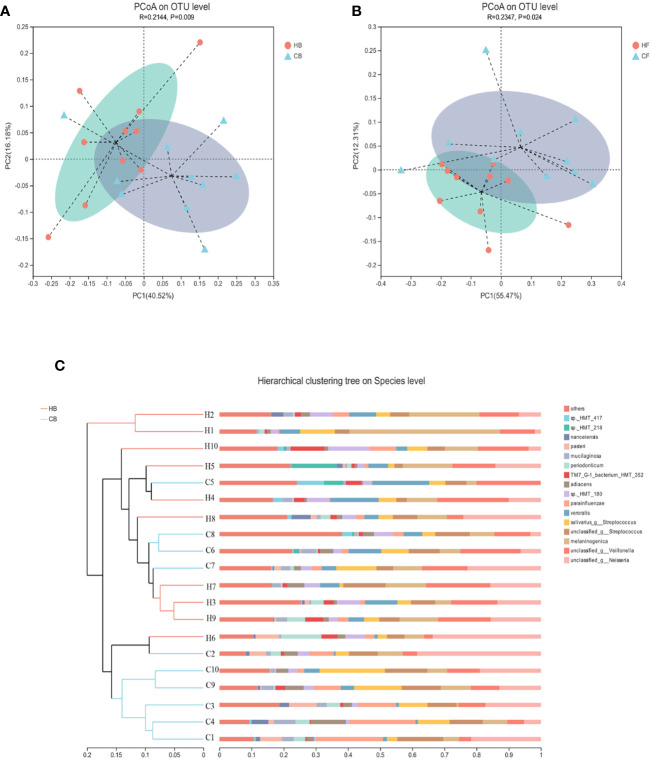
The inter-individual variations of the microbiome community structure. **(A, B)** The PCoA based on the weighted normalized UniFrac distance present the intel-individual variations of the tongue coating microbiota in the different groups. **(C)** The hierarchical clustering analysis (HCA) based on the weighted normalized UniFrac distance for baseline samples.

The similarities of the bacterial communities between the halitosis and control groups were compared by ANOSIM, based on the weighted normalized UniFrac distance, and visualized by PCoA plots. The ANOSIM analysis revealed significant differences in the structure of the tongue coating microbiota between halitosis and healthy groups, both at baseline and at follow-up (**Figures 4A, B**; *P* = 0.020 and *P* = 0.005, respectively).

The hierarchical clustering analysis (HCA) based on the weighted normalized UniFrac distance revealed that the HB, and CB groups have different microbiota community structures (**Figure 4C**). There was a tendency for the bacterial profiles to separate before the onset of the disease.

### Predictive Model for Halitosis Detection Based on Tongue Coating Microbiome Biomarkers

**Figure 5:**
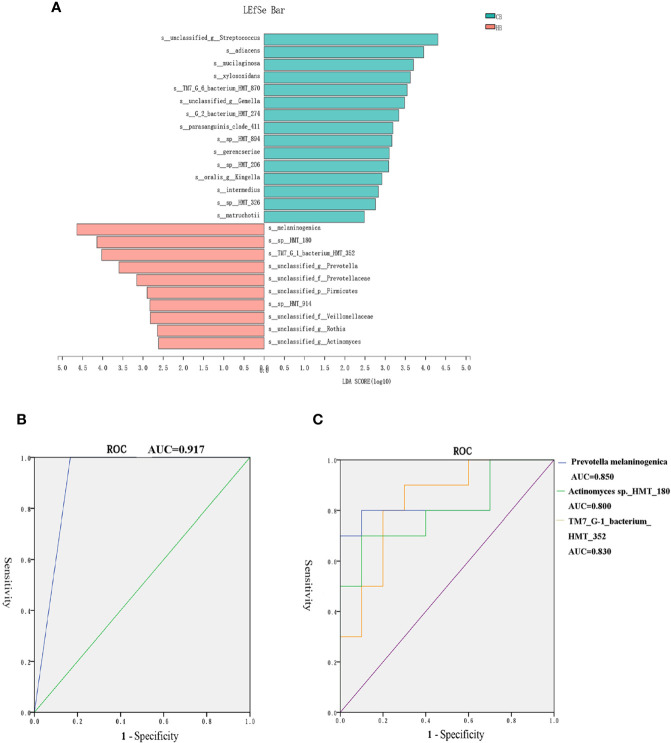
Biomarkers in the tongue coating microbiome prior to the onset of halitosis. **(A)** Microbiome alterations at the species level in the early stage. Comparison of the relative bacterial abundances on the species level using the LDA effect size (LEfSe) algorithm between the HB and CB groups. **(B)** The ROC for the prediction model. **(C)** The ROC curves for the final three biomarkers.

The LDA effect size (LEfSe) algorithm was applied for biomarker discovery prior to the onset of halitosis in children. The comparison of the relative bacterial abundances in the HB and CB groups revealed a number of halitosis-enriched species at the early stage. The microbiome in the HB group was characterized by 10 microbial biomarkers, including *Actinomyces* sp.*_HMT_180*, *Prevotella melaninogenica* and *Saccharibacteria TM7_G-1_bacterium_HMT_352* (LDA Score (log10) > 2), while bacterial species, such as *Granulicatella adiacens*, were enhanced in abundance among healthy children, when compared to those who developed halitosis over the period of 12 months (**Figure 5A**). The LEfSe results for the HF and CF groups are presented in **Appendix Figure 3**.

In order to eliminate the interference of interactions among biomarkers, further Spearman’s correlation analyses were carried out to explore the correlation of the relative abundance of various biomarkers. The heatmap of species correlations demonstrated that there was no significant correlation among these biomarkers (**Appendix Figure 4**).

Based on the LefSe results, a logistic regression model for halitosis prediction was generated using the relative abundance information of the biomarkers. After screening, *Actinomyces* sp.*_HMT_180, Prevotella melaninogenica* and *SaccharibacteriaTM7_G-1_bacterium_HMT_352* were finally included in the model.

**Figure 5B** presents the receiver operating characteristic (ROC) curve for the prediction model. The area under curve (AUC) for the model ROC curve was 0.917. The AUC values of the final three biomarkers, *Prevotella melaninogenica*, *Actinomyces* sp.*_HMT_180* and *Saccharibacteria TM7_G-1_bacterium_HMT_352*, were 0.850, 0.800 and 0.830, respectively (**Figure 5C**).

### The Differential Metabolism Capability Correlated to Halitosis

**Figure 6:**
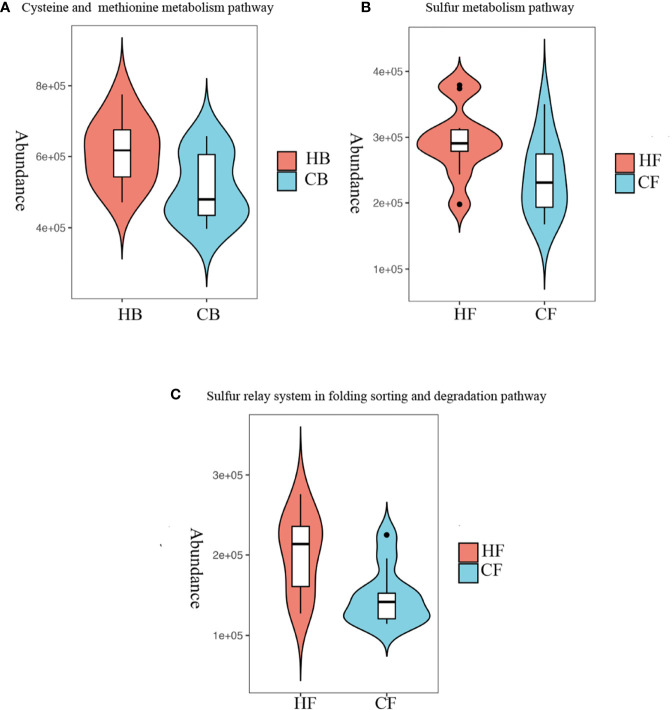
Metabolism alterations in the tongue coating microbiome at the early stage. Predicted functional profiling of microbial communities by PICRUSt, which collapsed into level 3 in the KEGG database pathways. **(A)** Comparison of the cysteine and methionine metabolism pathway between the HB and CB groups; *P* = 10.028. **(B)** Comparison of the sulfur metabolism pathway between the HF and CF groups; *P* = 10.039. **(C)** Comparison of the sulfur relay system in the folding, sorting and degradation pathway between the HF and CF groups; *P* = 10.012.

In order to characterize the functional alterations in terms of tongue coating microbiome in halitosis, the functional composition profiles were determined for all samples using the 16S rRNA sequencing data with PICRUSt, with particular focus on sulfur metabolism (**Figure 6**).

At baseline, the multiple KEGG (level 3) categories indicated that the cysteine and methionine metabolism pathway exhibited a higher level in the HB group, when compared to the CB group (*P* = 0.028). At the 12-month follow-up, the sulfur metabolism and sulfur relay system in the folding, sorting and degradation pathway suggested that there were more active metabolisms in the HF group, when compared to the CF group (*P* = 0.039 and *P* = 0.012, respectively). The heatmaps of all pathways (level 3) are shown in **Appendix Figure 5**.

The present study observed no differences in the metabolism of tryptophan, arginine, proline and lysine, which may result in halitosis by producing skatole, cadaverine, and putrescine (HB vs. CB *P* = 0.353, 0.159, 0.375; HF *vs.* CF *P* = 0.107, 0.421, 0.065, respectively). These findings suggest that halitosis in children may mainly attribute to these volatile sulfide compounds.

## Discussion

The present longitudinal study investigated the dynamic shift in the tongue coating microbial composition of halitosis-associated bacterial species during the disease progression among preschool children. For this purpose, 16S rRNA gene sequencing analysis and the Illumina Miseq platform were used to periodically evaluate the changes in tongue coating microbiome over the period of one year, and determine whether the participants would develop halitosis. The results of the present study revealed significant differences in the relative abundance of specific bacteria, microbial taxa and structural shifts prior to the development of halitosis. On the basis of such differences, a halitosis-onset prediction model was constructed. The findings emphasized the significance of the tongue coating microbiome for halitosis pathogenesis. Such shifts in the tongue coating microbiota prior to halitosis onset may predict the likelihood of disease onset, and assist in the early diagnosis among preschool children.

The human microbiota flora drastically varies depending on the harbor and distinct habitats (C. Human Microbiome Project, 2012). For example, there are distinct differences when comparing the microbiome characteristics of supragingival plaques associated with dental caries (Ling et al., 2013) and subgingival plaques for periodontitis (Abusleme et al., 2013). The tongue coating microbiome has been extensively investigated for halitosis studies (Simon-Soro et al., 2013; Ren et al., 2016a; Seerangaiyan et al., 2017). The anatomical deep fissures on the tongue surface has low oxygen potential, which facilitates a relatively anaerobic niche, and the colonization of pathogens associated with halitosis (Seerangaiyan et al., 2018). Therefore, investigating the changes in tongue coating microbiota during the development of halitosis may enhance the further understanding of the pathoetiology.

Previously, the investigators reported that the accumulation of bacterial plaque and poor oral hygiene are the main risk factors for halitosis in adults (Chen et al., 2016). Furthermore, poor oral hygiene, dry mouth and periodontal diseases (such as periodontitis and gingivitis) are the main contributory factors for oral halitosis (Amir et al., 1999; Lin et al., 2003; Motta et al., 2011; Yilmaz et al., 2012; Villa et al., 2014). The present study included preschool children, considering that this age group comprises of nonsmokers, and rarely report of having any periodontal lesion, dry mouth, or systemic diseases. This makes these group clinically suitable candidates for investigating the natural progression of halitosis, and the shift of the associated microbiomes. In addition, the primary dentition is considered relatively stable in this age group, since the eruption of permanent dentition would not be there. Furthermore, the eruption of permanent teeth may alter the oral microbiome (Shi et al., 2016; Shi et al., 2018), which can contribute to the progression of the disease in children. Since the present study included preschool children who share similar daily nutrients and oral hygiene care during the daytime, the impact of permanent teeth eruption on the tongue coating microbiome can be ignored. Accordingly, the data from the questionnaires revealed no significant differences in the diet and living habits of children, when compared to the halitosis and control groups. Based on these factors, the present longitudinal study can be considered as a well-controlled study.

The investigators reported a decrease in bacterial diversity (Shannon) and an increase in richness (Chao) after halitosis onset (**Figure 2**), suggesting that although the number of bacteria increased during the halitosis progression, the bio-distribution become more uneven. A previous study conducted by Dashper *et al*. also reported the increase in taxa number among children within 1.90-48.60 months old (Dashper et al., 2019). In contrast, a longitudinal study investigated the dental caries in three-year-old children, and reported no significant differences in the Chao index and Shannon index during the progression of the disease (Xu L. et al., 2018).

Before the onset of halitosis and its clinical manifestation, both the HB and CB groups carried distinct microbial communities that differed not only in the microbial structure at baseline, but also in shift patterns during the 12-month period (**Figures 3** and **4**). These dynamic differences were attributed to the persistent host modulation, that is, the major main factor that determines the structural, functional and ecological aspects of the microbial community (Baker et al., 2017). At present, literature on the development of oral microbiome among children remains scarce. Few longitudinal studies (Gomez and Nelson, 2017; Dzidic et al., 2018; Xu L. et al., 2018) have compared the oral microbiome and age-related dissimilarities on microbial oral diseases, such as early children caries.

A longitudinal study conducted on 3-year-old children reported no remarkable shift in the supragingival microbiome among healthy children after the 12-month follow-up period (Xu L. et al., 2018). Considering the relatively short observation time and stable primary dentition in the present study, the shifts of the tongue coating microbiome in the halitosis groups can be mainly attributed to the change in disease status.

The difference in certain microbial taxa may indicate the established pathologies and early warning signs (Teng et al., 2015). Therefore, children in the control and halitosis groups were further evaluated in terms of compositional differences at the species-level using LefSe for biomarker discovery. *Prevotella melaninogenica* and *actinomyces* sp.*_HMT_180* were found to be significantly more abundant in both the HB and in HF groups, when compared to the controls (**Figure 5**, **Appendix Figure 2**).

*Prevotella melaninogenica* is an anaerobic bacterium that is abundant in the upper respiratory tract flora, and associated with various anaerobic and mixed infections (Kondo et al., 2018). *Prevotella* is directly associated with VSC gas parameters, and is considered as the main contributory pathogen for halitosis (Tanaka et al., 2003; Scully and Greenman, 2008; Bollen and Beikler, 2012; Yang et al., 2013). The investigators previously reported that *Prevotella* may be the major organism that produces H_2_S in abundance in periodontally healthy adults suffering from halitosis (Ye et al., 2019). These findings are consistent with the study conducted by Riggio *et al*., which also identified *Prevotella melaninogenica* as the most commonly found species in the halitosis group (Riggio et al., 2008). Ademovski et al. conducted a randomized controlled clinical trial while treating the tongue microbiota of halitosis patients, and reported a significant reduction in *Prevotella melaninogenica* in halitosis participants after 14 days of treatment (Ademovski et al., 2013). At the molecular level, *Prevotella* metabolizes various proteins and peptides into amino acids, which further biodegrades into sulfur compounds, short-chain fatty acids, ammonia and indole/skatole, contributing to the progression of periodontitis and halitosis (Kazor et al., 1999; Takahashi, 2015). Although the exact role of *Actinomyces* in halitosis is not fully understood, *Actinomyces graevenitzii* has been reported to be present in the tongue biofilm, and contribute to the halitosis (Bernardi et al., 2020). Similarly, *Actinomyces sp*. has exhibited a different relative abundance, when compared to the supragingival plaque microbiota of healthy and halitosis children (Ren et al., 2016b). Although the present study predicted the participation of *Prevotella melaninogenica* and *Actinomyces* sp.*_HMT_180* in the metabolism of sulfur and cysteine, and methionine metabolism, the role in halitosis pathogenesis needs to be further investigated.

On the basis of the differential biomarkers between the HB and CB groups, the investigators constructed a halitosis onset prediction model. At present, the main emphasis of the microbial disease is on the aetiopathogenesis of diseases, including dental caries and periodontitis. Further evidences have suggested that a shift in the microbiota precedes the disease onset (Yassour et al., 2016). In addition, the relative abundance pattern of metabolic pathways may suggest various healthy or pathological conditions (Rudney et al., 2015; Xiao et al., 2016). The present study validates these findings, and suggests that halitosis can be detected well before the clinical manifestation, which is the result of the shift of the tongue coating microbiome. In addition, the shift of the tongue coating microbiome can potentially be used for predicting halitosis in the early stage, and can be used as a valuable tool to design individualized therapies and better prognoses.

In addition, the microbiome metabolic pathways analysis revealed the abundance of pathways for the metabolism of cysteine and methionine in the HB group, which transforms amino acids into H_2_S (Xu L. et al., 2018; Dzidic et al., 2018). These findings are in line with earlier studies that reported that the main cause of oral halitosis is the microbial degradation of methionine and cysteine (Spencer et al., 2007; Scully and Greenman, 2012).

There are a few limitations and corresponding future research directions in the present study. First, the prediction model requires further research and validation using clinical samples. A validation group was not set when the study was designed. Second, the present study merely included 20 participants. The small sample size may limit the extrapolation of the findings. Therefore, further studies investigating a larger sample size, various age groups, validation groups, and repeated analysis can further verify the existing data to support the proposed model. Third, the PICRUSt predictions using the 16S marker gene sequence has its own limitations (Langille et al., 2013). Metagenomics can be used in further studies to explore the halitosis-related role in the micro-ecological environment.

## Conclusion

In summary, the present study reported that the tongue coating microbiota shifts during the progression of halitosis. The microbiome composition and relative abundance of the tongue coatings in the halitosis and health group remarkably differed, even prior to the onset of the clinical manifestations of halitosis. The halitosis prediction model constructed based on tongue coating microbiome biomarkers indicated the microbial shifts before the onset of the disease. Therefore, this can be utilized for the timely diagnosis and intervention of halitosis in children. The evaluation of the tongue coating microbiome biomarkers may assist in predicting the risk of halitosis in children. Accordingly, as a preventive measure, the tongue coating plaque control instructions can be provided to the parents or guardians prior to the onset of halitosis.

## Data Availability Statement

The datasets presented in this study can be found in online repositories. The names of the repository/repositories and accession number(s) can be found in the article/**Supplementary Material**.

## Ethics Statement

The studies involving human participants were reviewed and approved by the Ethics Committee at the Ninth People’s Hospital, School of Medicine, Shanghai Jiao Tong University, Shanghai, China. The patients/participants provided their written informed consent to participate in this study.

## Author Contributions

YZ and XC completed the study design, sample collection, clinical examination, data analysis and statistics, and interpretation, and drafted and critically revised the manuscript. CZ completed data analysis and statistics, and drafted and critically revised the manuscript. GC and JZ completed the sample collection and drafted and critically revised the manuscript. XF contributed to designed the study and drafted and critically revised the manuscript. All authors gave final approval and agree to be accountable for all aspects of the work. All authors contributed to the article and approved the submitted version.

## Conflict of Interest

The authors declare that the research was conducted in the absence of any commercial or financial relationships that could be construed as a potential conflict of interest.
