# Supplementary material for: Dynamic Alterations of Oral Microbiota Related to Halitosis in Preschool Children
Source: Front Cell Infect Microbiol. 2021 Feb 26;11:599467. doi: 10.3389/fcimb.2021.599467 (PMC7952759; doi:10.3389/fcimb.2021.599467)
Supplement: Supplementary file 1 [file DataSheet_1.docx]

**Supplementary material**

**Dynamic alterations of oral microbiota related to halitosis in preschool children**

**Yu Zhang , Ce Zhu, Guizhi Cao, Jingyu Zhan, Xiping Feng, Xi Chen**

**Appendix Figure 1 Information of sequencing data.** (A) The rarefaction curves of all the samples (*N*=40). The shape of the rarefaction curves demonstrate the complete plateau, suggesting that the sequencing results may reflect the microbial information. (B) The Venn diagram depicts the shared and unique OTU distributions of the four groups.

**Appendix Figure 2 The distribution of bacterial relative abundances of the four groups.** The community histograms demonstrate the bacterial relative abundance at various levels: (A) phylum, (B) class, (C) order, (D) family, (E) genus, and (F) species.

**
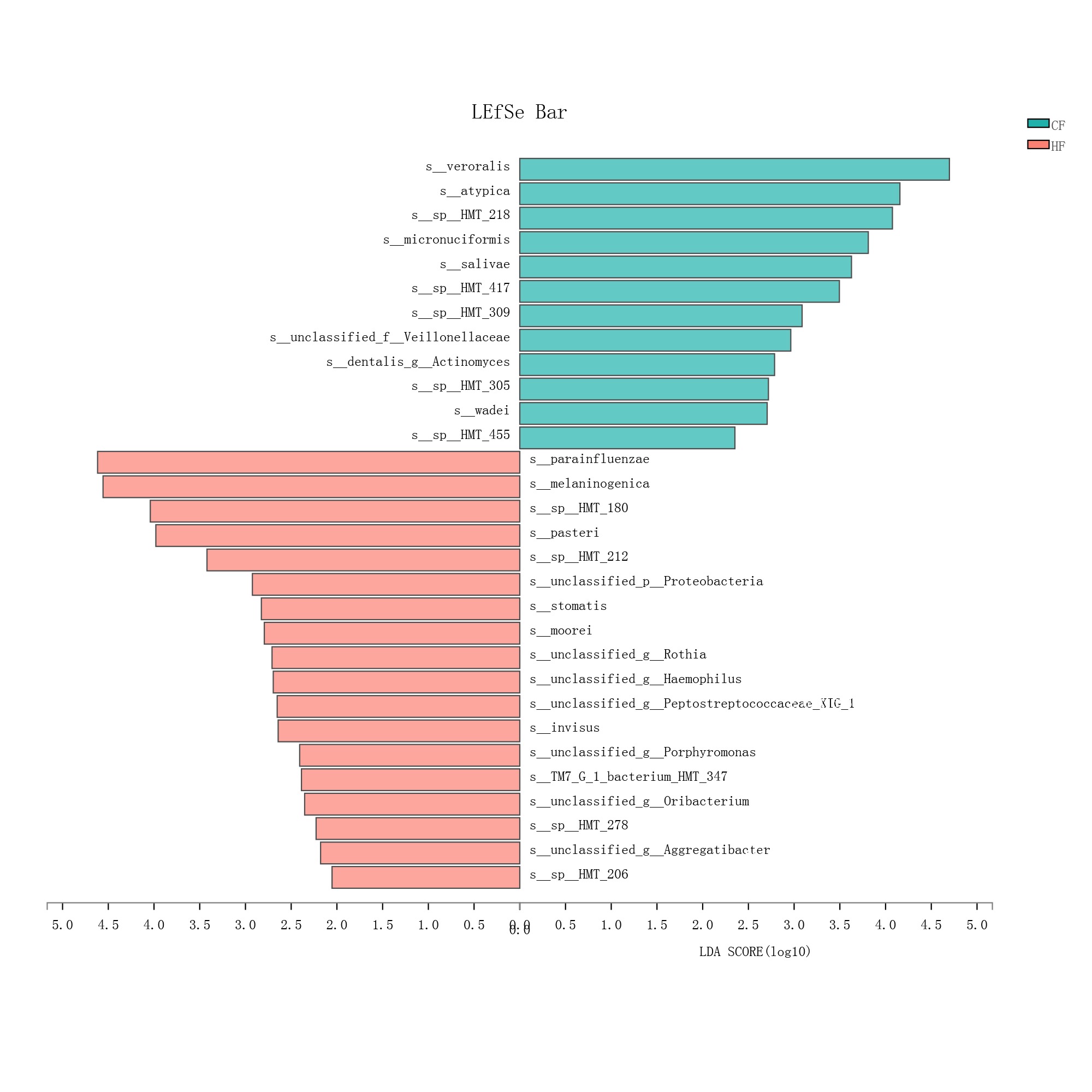
Appendix Figure 3 Microbiome alterations at the species level at the 12-month follow-up.** Comparison of relative bacterial abundances at the species level using the LDA effect size (LEfSe) algorithm between the HF and CF groups.

**Appendix Figure 4** **Correlation analyses of the relative abundance (%) of the most abundant species.**

**
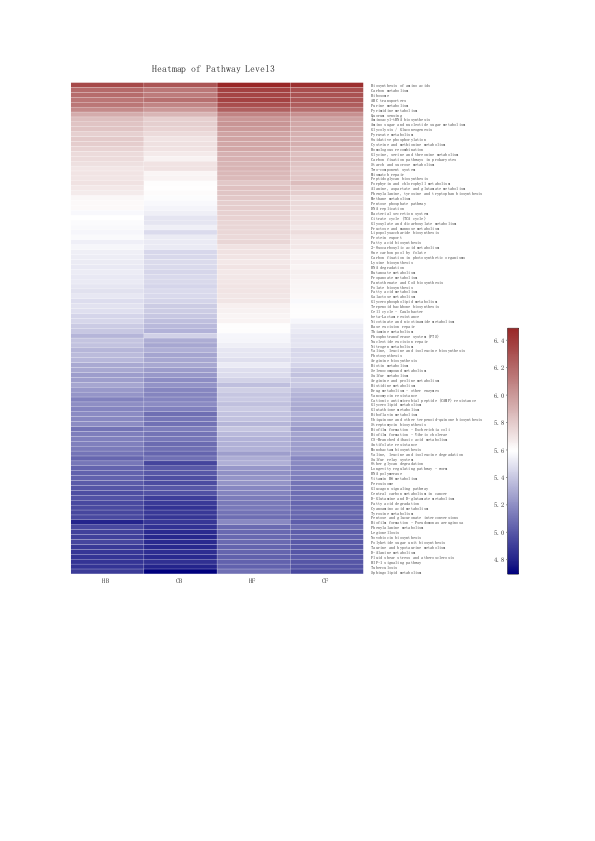
Appendix Figure 5 Heatmaps for all the predicted functional pathways.** The predicted functional profiling of microbial communities by PICRUSt, which collapsed into level 3 in the KEGG database pathways.

**Appendix Table 1. Demographic characteristics and health behaviours of the participants**

|  |  | Halitosis | Control | *P-values* |
| --- | --- | --- | --- | --- |
|  |  | N (%) | N (%) |  |
| Baseline | Age (months) |  |  | 0.545 |
|  | Mean ± SD | 41.10±5.26 | 42.40±4.09 |  |
|  | Gender |  |  | 1.000 |
|  | Man | 5 (50) | 5 (50) |  |
|  | Female | 5 (50) | 5 (50) |  |
|  | BMI (kg/m^2^) |  |  | 0.087 |
|  | Mean ± SD | 16.46±1.71 | 15.38±0.67 |  |
|  | Fruit intake |  |  | 0.305 |
|  | ≥1×/day | 9（90） | 0（0） |  |
|  | 1-6×/week | 1（10） | 10（10） |  |
|  | Seldom/never | 0（0） | 0（0） |  |
|  | Marmalade and honey intake |  |  | 0.582 |
|  | ≥1×/day | 0（0） | 0（0） |  |
|  | 1-6×/week | 1（10） | 3（30） |  |
|  | Seldom/never | 9（90） | 7（70） |  |
|  | Dessert intake |  |  | 0.063 |
|  | ≥1×/day | 2（20） | 6（60） |  |
|  | 1-6×/week | 8（80） | 4（40） |  |
|  | Seldom/never | 0（0） | 0（0） |  |
|  | Candy intake |  |  | 0.069 |
|  | ≥1×/day | 0（0） | 4（40） |  |
|  | 1-6×/week | 8（80） | 4（40） |  |
|  | Seldom/never | 2（20） | 2（20） |  |
|  | Beverage intake |  |  | 0.211 |
|  | ≥1×/day | 0（0） | 0（0） |  |
|  | 1-6×/week | 0（0） | 3（30） |  |
|  | Seldom/never | 10（100） | 7（70） |  |
|  | Any sweet snack intake |  |  | 0.063 |
|  | ≥1×/day | 2（20） | 6（60） |  |
|  | 1-6×/week | 8（80） | 4（40） |  |
|  | Seldom/never | 0（0） | 0（0） |  |
|  | Animal viscera intake |  |  | 0.582 |
|  | ≥1×/day | 0（0） | 0（0） |  |
|  | 1-6×/week | 1（10） | 3（30） |  |
|  | Seldom/never | 9（90） | 7（70） |  |
|  | Snack intake before sleeping* |  |  | 0.981 |
|  | ≥1×/day | 3（30） | 3（33.3） |  |
|  | 1-6×/week | 1（10） | 1（11.1） |  |
|  | Seldom/never | 6（60） | 5（55.6） |  |
|  | Regular oral examination within six months |  |  | 0.549 |
|  | Twice or more | 0（0） | 1（10） |  |
|  | Once | 3（30） | 2（20） |  |
|  | Seldom/never | 7（70） | 7（70） |  |
|  | Tooth brushing frequency |  |  | 0.135 |
|  | ≥2×/day | 9（90） | 5（50） |  |
|  | 1×/day | 1（10） | 4（40） |  |
|  | Not everyday | 0（0） | 1（10） |  |
|  | Assistance in tooth brushing |  |  | 0.453 |
|  | Always | 9（90） | 7（70） |  |
|  | Sometimes | 1（10） | 2 (20) |  |
|  | Never or seldom | 0（0） | 1（10） |  |
| Follow-up | BMI (kg/m^2^) |  |  | 0.053 |
|  | Mean ± SD | 16.60±1.78 | 15.31±0.70 |  |
|  | Fruit intake |  |  | 0.589 |
|  | ≥1×/day | 9（90） | 8（80） |  |
|  | 1-6×/week | 1（10） | 1（10） |  |
|  | Seldom/never | 0（0） | 1（10） |  |
|  | Marmalade and honey intake |  |  | 0.305 |
|  | ≥1×/day | 9（90） | 0（0） |  |
|  | 1-6×/week | 1（10） | 10（10） |  |
|  | Seldom/never | 0（0） | 0（0） |  |
|  | Dessert intake |  |  | 0.295 |
|  | ≥1×/day | 3（30） | 4（40） |  |
|  | 1-6×/week | 3（30） | 5（50） |  |
|  | Seldom/never | 4（40） | 1（10） |  |
|  | Candy intake |  |  | 0.349 |
|  | ≥1×/day | 1（10） | 1（10） |  |
|  | 1-6×/week | 2（20） | 5（50） |  |
|  | Seldom/never | 7（70） | 4（40） |  |
|  | Beverage intake |  |  | 0.513 |
|  | ≥1×/day | 1（10） | 0（0） |  |
|  | 1-6×/week | 1（10） | 2（20） |  |
|  | Seldom/never | 8（80） | 8（80） |  |
|  | Any sweet snack intake |  |  | 0.295 |
|  | ≥1×/day | 3（30） | 4（40） |  |
|  | 1-6×/week | 3（30） | 5（50） |  |
|  | Seldom/never | 4（40） | 1（10） |  |
|  | Animal viscera intake |  |  | 1.000 |
|  | ≥1×/day | 0（0） | 0（0） |  |
|  | 1-6×/week | 1（10） | 1（10） |  |
|  | Seldom/never | 9（90） | 9（90） |  |
|  | Snack intake before sleeping* |  |  | 0.869 |
|  | ≥1×/day | 2（20） | 2（22.2） |  |
|  | 1-6×/week | 2（20） | 1（11.1） |  |
|  | Seldom/never | 6（60） | 6（66.7） |  |
|  | Regular oral examination within six months |  |  | 0.865 |
|  | Twice or more | 3（30） | 2（20） |  |
|  | Once | 2（20） | 2（20） |  |
|  | Seldom/never | 5（50） | 6（60） |  |
|  | Tooth brushing frequency |  |  | 0.478 |
|  | ≥2×/day | 7（70） | 5（50） |  |
|  | 1×/day | 3（30） | 4（40） |  |
|  | Not everyday | 0（0） | 1（10） |  |
|  | Assistance in tooth brushing |  |  | 0.453 |
|  | Always | 9（90） | 7（70） |  |
|  | Sometimes | 1（10） | 2 (20) |  |
|  | Never or seldom | 0（0） | 1（10） |  |

*P*-values: Obtained by Chi-square test, Student’s *t*-test (two groups), or Fisher's exact test. *Some data are missing for these variables. SD: standard deviation. BMI: body mass index.
